# Comparison of Plasma Polymerized Thin Films Deposited from 2-Methyl-2-oxazoline and 2-Ethyl-2-oxazoline: II Analysis of Deposition Process

**DOI:** 10.3390/ijms26178641

**Published:** 2025-09-05

**Authors:** Peter Papp, Věra Mazánková, Ladislav Moravský, Ján Blaško, Pavel Sťahel, Lubomír Prokeš, Radek Horňák, Marián Lehocký, Hana Pištěková, David Trunec

**Affiliations:** 1 Department of Experimental Physics, Faculty of Mathematics, Physics and Informatics, Comenius University in Bratislava, Mlynská Dolina F2, 842 48 Bratislava, Slovakia; peter.papp@fmph.uniba.sk (P.P.); ladislav.moravsky@fmph.uniba.sk (L.M.); jan.blasko@fmph.uniba.sk (J.B.); 2 Department of Mathematics and Physics, Faculty of Military Technology, University of Defence in Brno, Kounicova 65, 662 10 Brno, Czech Republic; vera.mazankova@unob.cz; 3 Department of Plasma Physics and Technology, Faculty of Science, Masaryk University, Kotlářská 2, 611 37 Brno, Czech Republic; pstahel@physics.muni.cz (P.S.); luboprok@gmail.com (L.P.); rhornak@physics.muni.cz (R.H.); 4 Centre of Polymer Systems, University Institute, Tomas Bata University in Zlín, Trida Tomase Bati 5678, 760 01 Zlin, Czech Republic; lehocky@utb.cz (M.L.); pistekova@utb.cz (H.P.)

**Keywords:** plasma polymer, 2-oxazoline, G4 ionization and bond dissociation energies, antibiofouling coatings

## Abstract

Poly(2-oxazoline) coatings with antibiofouling properties and good biocompatibility can also be deposited by the plasma polymerization method using 2-methyl-2-oxazoline and 2-ethyl-2-oxazoline as monomers. Plasma polymers are formed of various monomer fragments and recombination products. Commonly, plasma polymers are highly crosslinked structures created by many different fragments, preferably of no repeating unit. Thus, chemical analysis of plasma polymers is difficult. To obtain a better description of plasma polymerized poly(2-oxazoline) coatings, the analysis of their plasma deposition process was performed. The electron ionization of 2-methyl-2-oxazoline and 2-ethyl-2-oxazoline molecules was studied using the crossed electron–molecular beam technique with mass spectrometric detection of the produced ions. The chemical composition of gaseous compounds at plasma polymerization was determined by gas chromatography-mass spectrometry (GC-MS), ion mobility spectrometry (IMS) and optical emission spectroscopy (OES). Also, the chemical composition and antibacterial activity of the water leachates from previously deposited poly(2-oxazoline) films were tested using FTIR spectroscopy and the disk diffusion method, respectively. It was found that acetonitrile and propionitrile are the main neutral products created in the nitrogen discharge with 2-methyl-2-oxazoline and 2-ethyl-2-oxazoline monomers. The water leachates from deposited films do not exhibit any antibacterial activity. It was concluded that the antibacterial properties of POx films are due to their hydrophility.

## 1. Introduction

Poly(2-oxazoline) (POx) coatings are an example of antibiofouling coatings [1], which could be used in biomedical applications and which could replace poly(ethylene glycol) (PEG) coatings used as hydrophilic antibiofouling coatings now. However, PEG coatings have some shortcomings, e.g., oxidative degradation or immunogenicity [2], so the study of POx is of great interest. POx coatings can be deposited using the plasma polymerization method, which is in some respects more useful than conventional polymerization methods (e.g., cationic ring-opening polymerization or grafting).

The plasma polymerization process can be carried out under various experimental conditions and plasma reactor configurations. Low-pressure discharges as well as atmospheric pressure discharges can be used. Bhatt et al. [3] used for plasma polymerization of POx from 2-ethyl-2-oxazoline a low pressure inductively excited pulsed radio frequency (RF) discharge. The group of Vasilev performed plasma deposition of 2-methyl-2-oxazoline and 2-ethyl-2-oxazoline in low pressure RF discharge [4,5,6,7].

In our previous studies, POx coatings were deposited on glass or teflon substrates using plasma polymerization with 2-methyl-2-oxazoline or 2-ethyl-2-oxazoline vapours as a monomer [8,9]. The plasma polymerization was performed in a volume dielectric barrier discharge (DBD) burning in nitrogen at atmospheric pressure. Our work was focused on the development of antibacterial layers with good cytocompatibility. The prepared POx coatings were tested for antibacterial activity according to ISO 22196:2011 standard [10]. Most of the coatings showed very strong antibacterial activity against both bacterial strains *S. aureus* (CCM 4516) and *E. coli* (CCM 4517) according to this standard. It was found that the coatings deposited from 2-methyl-2-oxazoline exhibited better antibacterial activity against *E. coli* and *S. epidermidis* bacterial strains and also exhibited better cytocompatibility.

Our analysis extends the contributions of earlier studies. This study aims to understand the differences between layers deposited from different monomers and to find out what causes the antibacterial properties of these layers. To understand the mechanisms behind the antibacterial properties of POx coatings, and the differences between films derived from 2-methyl-2-oxazoline and 2-ethyl-2-oxazoline, we conducted an in-depth analysis of their plasma deposition processes. Several diagnostic methods were selected, namely crossed electron–molecular beam technique with mass spectrometric detection, gas chromatography–mass spectrometry (GC-MS), optical emission spectroscopy (OES) and ion mobility spectroscopy–mass spectrometry (IMS-MS). The previously prepared layers were also extracted in water and the solutions were analysed using Fourier-transform infrared spectroscopy (FTIR) and antibacterial tests. The crossed electron–molecular beam technique with mass spectrometric detection was used for the study of electron ionization of 2-methyl-2-oxazoline and 2-ethyl-2-oxazoline molecules. Energy resolved mass spectra and electron energy dependent ion efficiency curves for ions of particular mass charge ratios (m/z) have been obtained. Except for the ionization energy of the 2-methyl-2-oxazoline and 2-ethyl-2-oxazoline molecules, the appearance energies of most of the ions in the mass spectrum formed via dissociative ionization (DI) processes have been determined. Quantum-chemical calculations of the neutral molecule, molecular ions, and neutral and ionic fragments have been carried out and the reaction enthalpies for the DI processes were calculated. The theoretical DI values were applied to identify the DI reactions and fragment ions in the experiment. GC-MS and OES were used to analyse gaseous products in the discharge. GC-MS technique enables to separate and detect the neutral products, created in the discharge by chemical reactions. OES detects the excited states of atoms and molecules in the discharge. IMS-MS was used for the analysis of monomers and the reaction products of monomers with $H_{3}$$O^{+}$ ions.

## 2. Results

### 2.1. Electron Ionization

The results for electron ionization of 2-methyl-2-oxazoline are shown in Table 1.

This table shows ions with the lowest appearance potential and the highest signal intensities. The highest signal intensities were observed for ions with masses of 55, 43 and 85 in decreasing order.

The results for electron ionization of 2-ethyl-2-oxazoline are shown in Table 2.

Again, this table shows ions with the lowest appearance potential and highest signal intensities. The highest signal intensities were observed for ions with masses of 69, 54 and 41 in decreasing order.

**Figure 1:**
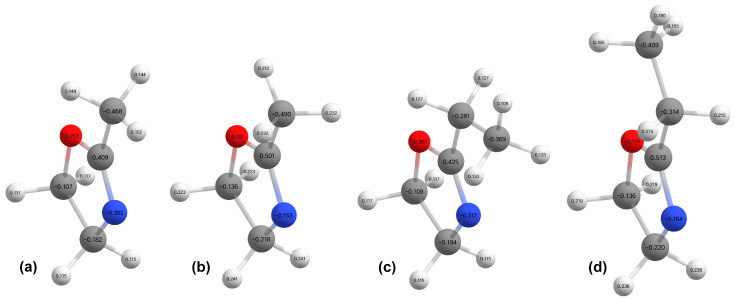
The B3LYP/GTBas3 optimal geometries with charges on atoms (hydrogens—white, carbons—grey, nitrogen—blue, oxygen—red) for (**a**) neutral and (**b**) cation 2-methyl-2-oxazoline, as well as (**c**) neutral and (**d**) cation 2-ethyl-2-oxazoline.

**Figure 2:**
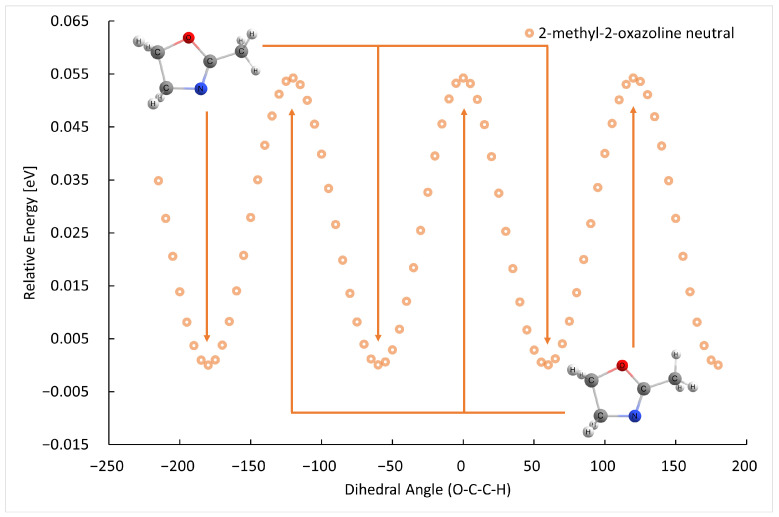
The B3LYP/GTBas3 potential energy surface scan as a function of O-C-C-H dihedral angle of neutral 2-methyl-2-oxazoline, with three identical ground state geometries labeled with arrows down, and three identical transition state geometries labeled with arrows up.

**Figure 3:**
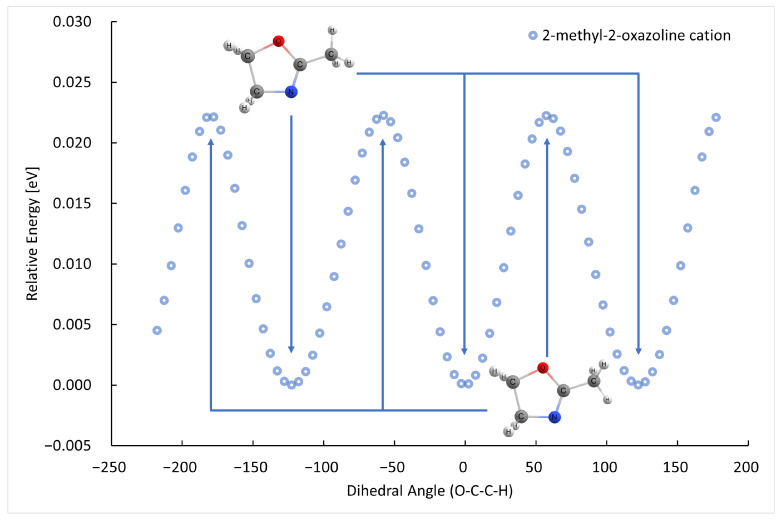
The B3LYP/GTBas3 potential energy surface scan as a function of O-C-C-H dihedral angle of cation 2-methyl-2-oxazoline, with three identical ground state geometries labeled with arrows down, and three identical transition state geometries labeled with arrows up.

**Figure 4:**
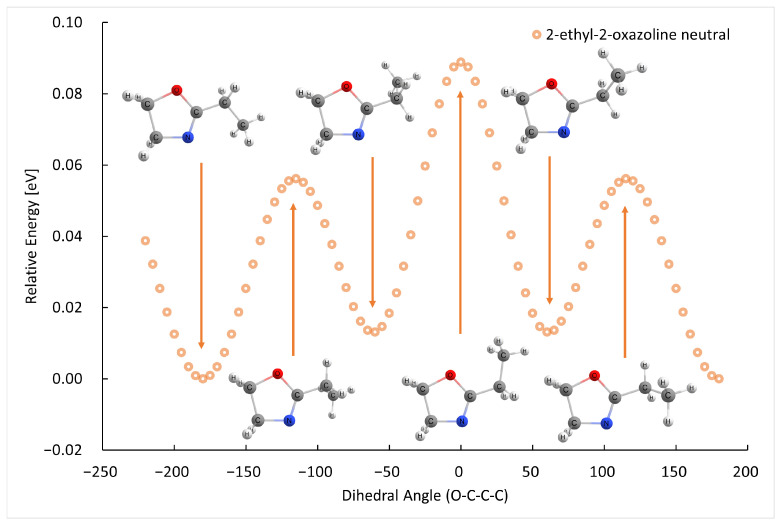
The B3LYP/GTBas3 potential energy surface scan as a function of O-C-C-C dihedral angle of neutral 2-ethyl-2-oxazoline, with one ground state geometry and two identical conformational isomers labeled with arrows down, and the corresponding transition state geometries labeled with arrows up.

**Figure 5:**
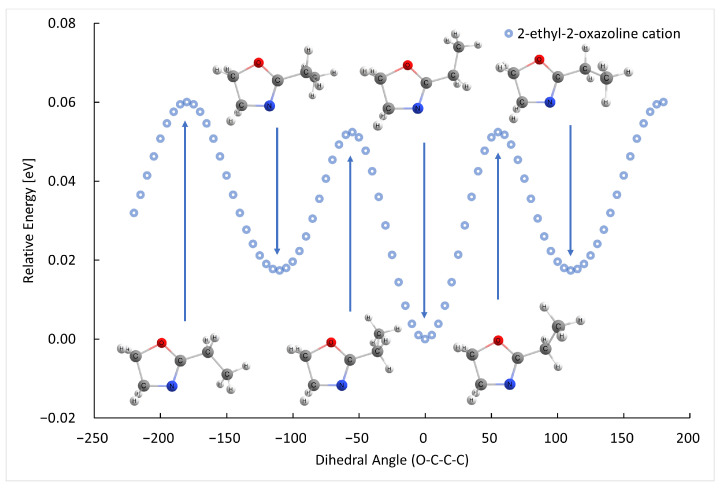
The B3LYP/GTBas3 potential energy surface scan as a function of O-C-C-C dihedral angle of cation 2-ethyl-2-oxazoline, with one ground state geometry and two identical conformational isomers labeled with arrows down, and the corresponding transition state geometries labeled with arrows up.

The B3LYP/GTBas3 optimal geometries of neutral and cation 2-methyl-2-oxazoline and 2-ethyl-2-oxazoline are in Figure 1. The optimal geometries were identified from a 1-dimensional potential energy scan as a function of dihedral angle O-C-C($H_{2}$)-H of 2-methyl-2-oxazoline, and O-C-C($H_{2}$)-C($H_{3}$) of 2-ethyl-2-oxazoline, plotted in Figure 2, Figure 3, Figure 4 and Figure 5 respectively. The ground state of neutral 2-methyl-2-oxazoline is A′1Cs symmetry, with one methyl hydrogen oriented towards the nitrogen atom and the remaining out of plane methyl hydrogens towards the oxygen atom. This is also valid for 2-ethyl-2-oxazoline where the hydrogen atom close to nitrogen is substituted with a methyl group, oriented towards the nitrogen atom, again with A′1Cs symmetry. The $180^{{}^{\circ}}$ rotation of the functional groups in 2-methyl or 2-ethyl-2-oxazoline around the C-C($H_{3}$) or C-C($H_{2}$) bond respectively, identified conformers, which are transition states along with the stabilisation of methyl hydrogen or the methyl group of ethyl towards the oxygen atom. For 2-ethyl-2-oxazoline, several other local minima and transition states are found with the potential energy scan, depicted in Figure 4. The ionization of both molecules retains the $C_{s}$ symmetry for both molecules, however, it changes the symmetry of the final ground state to A″2, together with the geometries of both cations. Contrary to the neutrals, the oxygen atom becomes more attractive for the in-plane hydrogen atom of the methyl group in 2-methyl-2-oxazoline and the remaining two out-of-plane methyl hydrogen atoms are oriented towards nitrogen, similarly for the whole ethyl group rotated by $180^{{}^{\circ}}$ with methyl oriented towards the oxygen. For further investigation of the thermodynamic thresholds of ionisation energies and appearance energies listed in Table 1 and Table 2 we used only the ground state energies, recomputed with the G4 method.

The results for dissociation energies of 2-methyl-2-oxazoline are shown in Table 3.

The results for dissociation energies of 2-ethyl-2-oxazoline are shown in Table 4.

### 2.2. GC-MS Measurements

**Figure 6:**
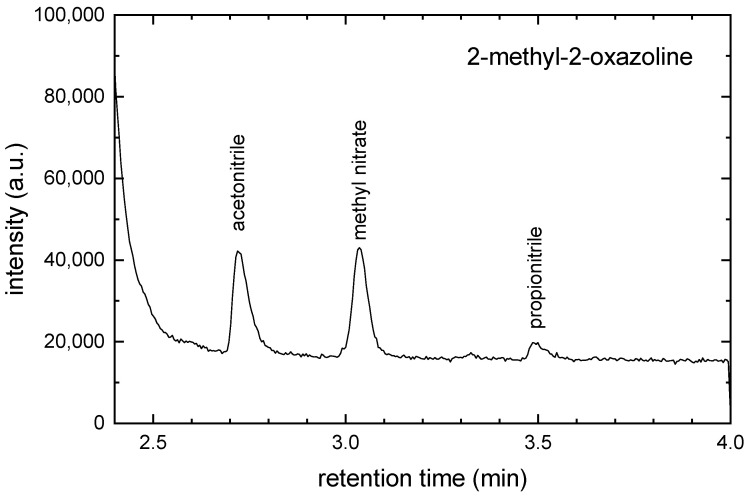
The GC-MS chromatogram for 2-methyl-2-oxazoline monomer.

The chemical composition of the gas in the discharge chamber was analyzed using GC-MS. The gas samples were trapped in a cold trap. Then the trap was heated and the gas from the trap was introduced to the GC. The GC-MS chromatogram for 2-methyl-2-oxazoline monomer is shown in Figure 6.

**Figure 7:**
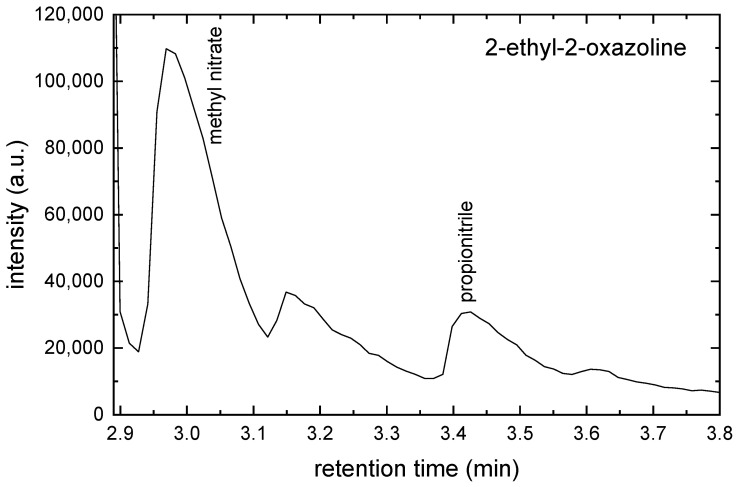
The GC-MS chromatogram for 2-ethyl-2-oxazoline monomer.

The acetonitrile (C$H_{3}$CN), methyl nitrate (C$H_{3}$N$O_{3}$) and propionitrile (C$H_{3}$C$H_{2}$CN) were identified in this chromatogram. The GC-MS chromatogram for 2-ethyl-2-oxazoline monomer is shown in Figure 7.

The methyl nitrate and propionitrile were identified in this chromatogram. The peak at a retention time of 3.15 min was not unambiguously identified.

### 2.3. Optical Emission Spectroscopy of Plasma

**Figure 8:**
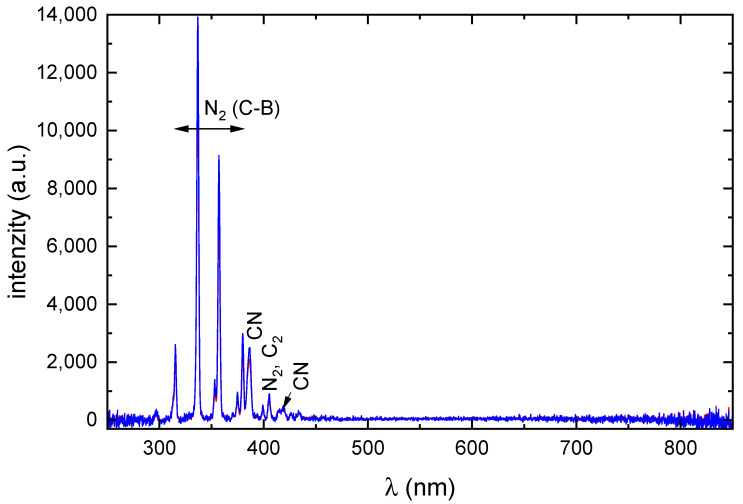
The optical emission spectra of the discharge with 2-oxazoline monomers. Blue line—2-methyl-2-oxazoline; red line—2-ethyl-2-oxazoline monomer.

The optical emission spectra of the nitrogen discharge with 2-methyl-2-oxazoline and 2-ethyl-2-oxazoline monomers are shown in Figure 8.

The spectra are dominated by molecular bands of $N_{2}$ and CN, while atomic hydrogen (Balmer series) and oxygen lines expected from monomer dissociation are absent. This absence is consistent with the electron energy distribution function (EEDF) in atmospheric-pressure nitrogen discharges, which drops sharply for electron energies above ∼10 eV, leaving few electrons energetic enough to excite Hα (12.1 eV) or O I 777 nm (10.7 eV) transitions. Similar observations have been reported by Snirer et al. [11], when these lines were also not recorded in their experiments particularly in regions of the discharge remote from the electrode nozzle.

### 2.4. IMS-MS Measurement

In air, the primary positive ions $N_{2}^{+}$ and $O_{2}^{+}$ are converted to $H^{+}$·(${H_{2}O)}_{n}$ ions by a chain of well-known reactions in wet air [12]. The ionisation of analyte M in APCI proceeds via the following reactions

(1) $$H^{+}\cdot (H_{2}{O)}_{3,4}+M⟶M\cdot H^{+}+\left(3,4\right)H_{2}O$$

(2) $$H^{+}\cdot (H_{2}{O)}_{n}+M⟶M\cdot H^{+}\cdot (H_{2}O)+\left(n-1\right)H_{2}O$$

**Figure 9:**
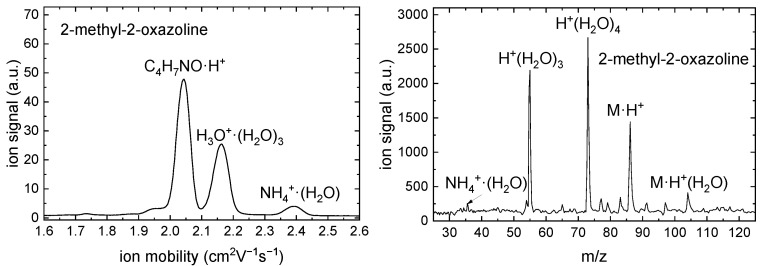
Positive polarity ion mobility spectrum (**left**) and mass spectrum (**right**) of the RI and 2-methyl-2-oxazoline.

**Figure 10:**
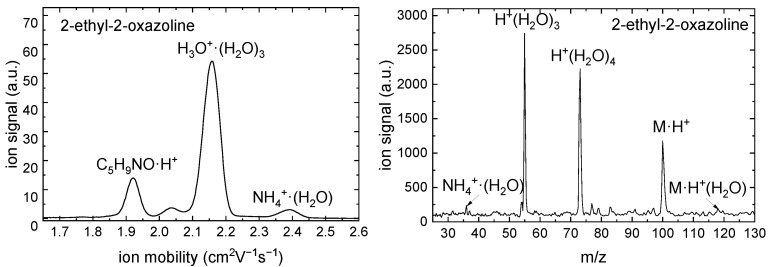
Positive polarity ion mobility spectrum (**left**) and mass spectrum (**right**) of the RI and 2-ethyl-2-oxazoline.

The results of IMS-MS measurements are shown in Figure 9 and Figure 10.

A monomer peak with the reduced ion mobility $K_{0}=2.046\;{\mathrm{cm}}^{2}V^{-1}s^{-1}$ was detected for 2-methyl-2-oxazoline in the IMS spectrum. The APCI of 2-methyl-2-oxazoline formed the ions with m/z 86 and 104 which correspond to M·$H^{+}$ and M·$H^{+}$·$H_{2}$O. A monomer peak with the reduced ion mobility $K_{0}=1.925\;{\mathrm{cm}}^{2}V^{-1}s^{-1}$ was detected for 2-ethyl-2-oxazoline in the IMS spectrum. The APCI of 2-ethyl-2-oxazoline formed the ions with m/z 100 and 118 which correspond to M·$H^{+}$ and M·$H^{+}$·$H_{2}$O. The peaks of N$H_{4}^{+}$·($H_{2}$O) ions correspond to APCI of ammonia, which is an impurity present in wet air used in the experiment. No impurities were detected in both oxazolines.

### 2.5. FTIR Analysis and Evaluation of Antibacterial Activity of Film Extract

**Figure 11:**
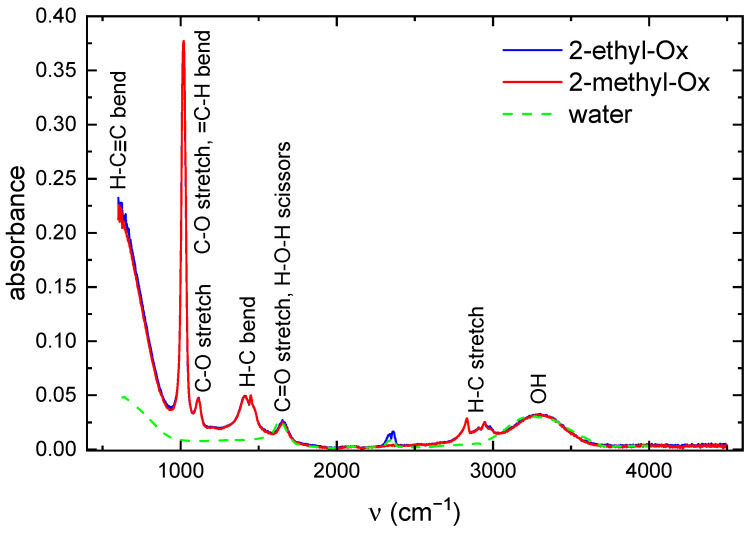
The FTIR spectrum of leachates from POx films (full lines) and pure water (green dashed line). The absorbance values for the water spectrum were multiplied by a factor of 0.2. The small peak at 2350 ${\mathrm{cm}}^{-1}$ is an instrument artefact.

The films deposited in previous studies [8,9] were immersed in water for 48 h. The FTIR spectra of leachates and pure water are shown in Figure 11.

Broad absorption band in the range 3000–3600 ${\mathrm{cm}}^{-1}$ consists of several peaks belonging to OH and NH groups. The bands at 2945 ${\mathrm{cm}}^{-1}$ and 1450 ${\mathrm{cm}}^{-1}$ are characteristic for vibrations of C$H_{3}$ and C$H_{2}$ groups. The band at 1660 ${\mathrm{cm}}^{-1}$ is characteristic for H-O-H bend (scissors) vibration and also for C=O stretch. The bands at 1015 ${\mathrm{cm}}^{-1}$ and 620 ${\mathrm{cm}}^{-1}$ can be assigned to bend vibrations in =C-H and C≡C-H groups respectively. If we compare these FTIR spectra with FTIR spectra of deposited films, we find that the NH and CN peaks presented in the FTIR spectra of films are not in the spectra of leaches. On the contrary the peaks at 1024 ${\mathrm{cm}}^{-1}$ and 600 ${\mathrm{cm}}^{-1}$ were not observed in the FTIR spectra of films.

**Figure 12:**
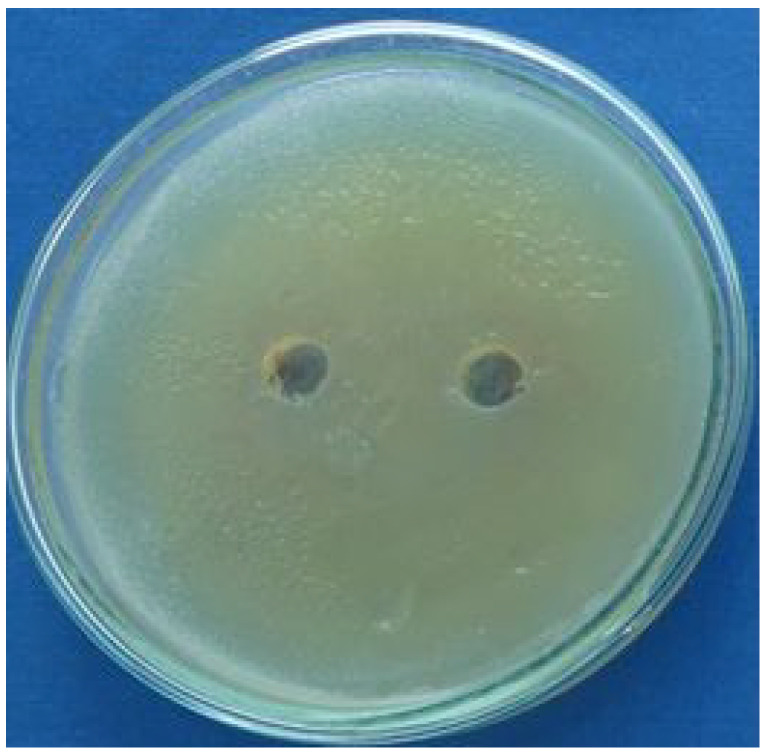
The result of the disk diffusion method for the leachate from the film deposited using 2-ethyl-2-oxazoline. The used bacterial strain was *E. coli*.

The film leachates were then tested for antimicrobial effect using the disk diffusion method. *Staphylococcus aureus* (CCM 4516) and *Escherichia coli* (CCM 4517) both supplied by the Czech Collection of Microorganisms in Brno were used for these tests. The result of such a test is shown in Figure 12.

No inhibition zone was observed in all cases. So, the leachates from the films do not eliminate the growth of the used bacterial strains.

## 3. Discussion and Conclusions

The nitrogen discharge properties will be briefly summarized as the POx coatings were deposited in discharges with nitrogen as working gas. Due to vibrational excitation of nitrogen by electrons and further inelastic collisions the electron energy distribution function drops sharply at electron energies higher than 10 eV and thus the number of electrons with energies higher than 10 eV is negligible [13]. For that reason, only ions with the lowest appearance energy can be considered. The metastable states of nitrogen A ∑u+3 and a Πg1 with energies 6.2 eV and 8.5 eV play important role in discharge mechanism [14]. Optical emission spectroscopy of the present discharges shows dominant molecular bands from $N_{2}$, CN, and $C_{2}$, with no detectable atomic hydrogen (Balmer series) or oxygen (O I 777 nm) lines. This absence can be explained by the high excitation thresholds for Hα (12.1 eV) and O I 777 nm (10.7 eV) lying well within the depleted region of the EEDF, combined with rapid collisional quenching at atmospheric pressure, which shortens the lifetimes of excited atomic species. As a result, free H and O atoms are either rare or radiatively suppressed in the observed plasma volume. Nevertheless, FTIR spectroscopy of the water-soluble fraction of the deposited films reveals clear C-O stretching modes, as well as =C-H, H-C bending, and H-C≡C vibrational features. This indicates that transient H and O radicals, produced during monomer dissociation, are efficiently incorporated into stable functional groups within the growing film, rather than remaining as detectable excited atoms in the gas phase.

When 2-oxazolines are added to nitrogen discharge, they quench the nitrogen metastable states. This quenching can lead to 2-oxazoline dissociation (see such reactions for methane [15]). The measured ionization energies of 2-methyl-2-oxazoline and 2-ethyl-2-oxazoline are 9.09 eV and 9.15 eV, respectively. To our knowledge, this is the first measurement of the ionization energy of these compounds. The ionization energies were also calculated using ab-initio quantum mechanical package GAUSSIAN 16. The ionization energies of 2-methyl-2-oxazoline and 2-ethyl-2-oxazoline are substantially lower than the ionization energy of molecular nitrogen (15.581 eV). In this case, the charge transfer reaction between molecular nitrogen ion $N_{2}^{+}$ and 2-oxazoline molecules is exothermic and can occur leading to 2-oxazoline ions. Also, the ionization of 2-oxazolines becomes the main process of charge production. The electron ionization of 2-oxazolines produces formaldehyde and formaldehyde radical (CH=O). This radical can react with an H atom in three body reaction producing formaldehyde again. The C=O bonds were identified in deposited films. The formaldehyde was not detected using the cold trap method, because gaseous formaldehyde polymerizes when condensed to a liquid. Further acetonitrile and propionitrile were produced at electron ionization of 2-methyl-2-oxazoline and propionitrile was produced at electron ionization of 2-ethyl-2-oxazoline. The acetonitrile and propionitrile are also produced in reactions of C$H_{2}$-C≡N and C$H_{2}$-C$H_{2}$-C≡N with H atom. The nitriles can be also produced by reactions in gas phase by reaction

(3) $$N{(}^{2}D)+H_{2}⟶\mathrm{NH}+H,$$

where ${N(}^{2}$D) is an excited nitrogen atom, and by following reaction

(4) $$\mathrm{NH}+C_{2}H_{3}⟶{\mathrm{CH}}_{3}C\equiv N+H,$$

which finally produces acetonitrile.

The propionitrile is also produced from C$H_{2}$-C≡N by reaction

(5) $${\mathrm{CH}}_{2}C\equiv N+{\mathrm{CH}}_{3}⟶{\mathrm{CH}}_{3}-{\mathrm{CH}}_{2}C\equiv N.$$

This reaction can explain the presence of propionitrile in the case of 2-methyl-2-oxazoline monomer. Acetonitrile and propionitrile were also detected as gaseous products from the discharge by GC-MS. The nitriles were also detected in the discharge in the mixture of nitrogen and hydrocarbons, see e.g., [16]. Stable products such as acetonitrile and propionitrile are probably not incorporated into the thin layer. However, experiments with electron ionization show that dissociative ionization also produces radicals such as C$H_{2}$-C≡N and C$H_{2}$${\mathrm{-CH}}_{2}$-C≡N, which can then be incorporated into the layers. The C≡N bond was also identified in deposited films. The methyl nitrate can be produced by the reaction of ${\mathrm{NO}}_{3}$ with C$H_{3}$. The C$H_{3}$ radical can be transformed by a chain of chemical reactions mainly with the H atom to $C_{2}$ and C atom, see [17]. The list of these reactions with their rate coefficients can be found in [18]. C atom can react with N atom in a three body reaction producing CN. Both CN and $C_{2}$ were observed in optical emission spectra. The difference in plasma deposition with 2-methyl-2-oxazoline and 2-ethyl-2-oxazoline monomers lies in the fact that the use of 2-ethyl-2-oxazoline monomer leads to fragments with longer carbon chains.

Acetonitrile and propionitrile are toxic; however, they are not antibacterial substances. Although the formaldehyde kills most bacteria, no antibacterial effect for film leachates was observed at the disk diffusion method. On the other hand, after rinsing the layers by water, their antibacterial activity against *S. epidermidis* decreased [8,9]. This would imply that the extracts from the layers should also be antibacterial. However, this is not the case, so the rinsing of the layers changes the layer morphology and also reduces their hydrophilicity. The water contact angles for POx films deposited using 2-methyl-2-oxazoline and 2-ethyl-2-oxazoline monomers are usually in range 10–$25^{{}^{\circ}}$, the total surface free energies are in range 42–57 ${\mathrm{mJ/m}}^{2}$ [8,9]. The preliminary experiments showed that the water contact angle of POx films after rinsing changed to the values in range 31–$33^{{}^{\circ}}$.

So, deposited POx films are reaching the limit of superhydrophilicity, which is characterized by a water contact angle of less than $10^{{}^{\circ}}$. In the aquatic environment, a protective hydration layer can be created on POx film surface due to its hydrophylic-polar nature, which generates hydration forces that prevent bacterial adhesion [19]. Also, the surface roughness of the substrate and deposited POx films differs, its value for deposited films can be higher or lower than the surface roughness of the substrate [20]. So, the antibacterial properties of POx films may result from their hydrophilic and smooth surface. Such a conclusion was also found for coatings deposited from pentane or hexane [21].

## 4. Materials and Methods

### 4.1. Materials

2-Methyl-2-oxazoline (purity≥97.5%) and 2-ethyl-2-oxazoline (purity ≥ 99.0%) (both from Sigma-Aldrich, Munich, Germany) were used as monomers for plasma deposition. Glass and teflon were used as substrates for deposition.

### 4.2. Plasma Reactor

**Figure 13:**
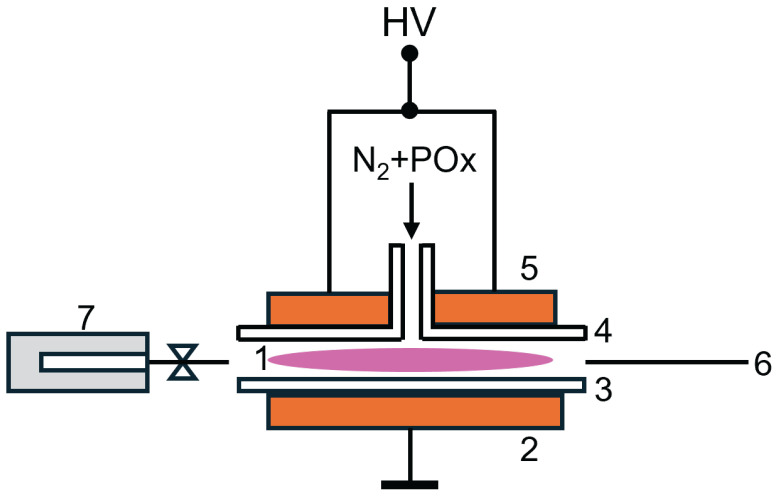
Experimental setup. 1—discharge, 2—bottom electrode, 3—substrate (glass), 4—dielectric (glass), 5—upper electrode, 6—optical fiber to spectrometer, 7—cold trap.

The schema of the plasma reactor used in this study is shown in Figure 13. The pictures of the plasma reactor and its detailed description can be found in previous papers [8,9].

This reactor was used for GC-MS and OES measurements. The dielectric barrier discharge (DBD) was generated between two planar metal electrodes. Both electrodes were covered with glass (thickness 1.5 mm). The discharge gap between the electrodes was set to 1.0 mm. The working gas (nitrogen) with the monomers (2-oxazolines) was supplied through a slit in the center of the upper electrode. The gas pressure in the plasma reactor was set to 101 kPa. The monomer vapors were supplied to the discharge using nitrogen flow bubbled through the liquid monomers. The total gas flow through the plasma reactor was 500 sccm. A high voltage with a frequency 6 kHz was used to generate a discharge in the discharge gap. The input power to a high voltage source was set to 55 W. The discharge was burning in a homogeneous atmospheric pressure Townsend discharge (APTD) mode [22]. Maximum electron density in such discharge was determined using a numerical model to be $10^{7}$ ${\mathrm{cm}}^{-3}$ [22]. The gas temperature in DBD is usually low, at experimental conditions described above the gas temperature was estimated (based on previous gas temperature measurement in coplanar DBD [23]) to be 40 °C.

The above described plasma reactor was used in previous studies [8,9] for deposition of POx coatings on glass substrates. The deposition time was 23 min. The obtained films have the thicknesses 0.6–1.7 μm, the film thickness decreases with increasing substrate temperature. Using nitrogen as a working gas for the discharge allows for obtaining a homogeneous mode of the discharge and to produce nitrogen-rich films. Nitrogen-rich surfaces are known for their excellent biocompatibility [24].

### 4.3. Electron Ionization Measurement

#### 4.3.1. Experiment

The electron ionization was studied using a crossed electron/molecular beams apparatus, CEMBIA [25,26,27,28,29,30]. The vapors of 2-oxazolines were introduced into the vacuum through a small capillary, forming an effusive molecular beam, which was crossed perpendicularly with an electron beam under single-collision conditions. The electron beam is generated with a trochoidal electron monochromator with an energy resolution of about 200 meV according to the measurement of the electron current dependency on the Farrady cup from the incident electron energy (the full width at half maximum of the obtained electron energy distribution function). The energy scale was calibrated according to the well-known appearance energy of ${\mathrm{Ar}}^{+}$ ion at 15.759 eV [31]. The charged products were extracted by a weak electric field into a quadrupole mass spectrometer Pfeiffer QMA400 (Balzers, Liechtenstein), with an extraction time approximately ∼ 10 μs and flight time through the quadrupole mass spectrometer ∼ 50 μs. The mass spectra were recorded at 70 eV, and the corresponding ion efficiency curves for a certain mass/charge ratio were acquired as a function of incident electron energy. The chosen electron energy of 70 eV is so high that all electrons in the DBD discharge have lower energy. The electron energy distribution function for electrons in nitrogen DBD decreases rapidly with increasing electron energy, so only the disociative ionization reactions with the lowest appearance energies will be significant in the discharge. The appearance energies (AE) were evaluated with a fitting procedure based on a Wannier formula [32,33,34,35] for the electron energy *E* dependency of the cross section σ(E) close to the threshold, convoluted with the electron energy distribution function (Gaussian function with FWHM 200 meV).

#### 4.3.2. Theory

The GAUSSIAN 16 programme package [36] was used to compute the ground state energies of the neutral and cationic 2-methyl-2-oxazoline and 2-ethyl-2-oxazoline, as well as their fragments after electron impact ionization. The composite G4 method [37] was used to obtain thermochemical data, ionization energies (EAs) and bond dissociation energies (BDEs) at room temperature with inclusion of zero-point vibrational corrections. The set of computed G4 enthalpies was used to derive the theoretical thermodynamic thresholds of the most relevant fragments identified from CEMBIA experiment and compared to the experimentally obtained appearance energies. The G4 method is a complex energy computation involving several pre-defined calculations on the specified molecular system with B3LYP [38,39] optimal geometries and frequencies. Higher accuracy is achieved with combination of several single point ab initio calculations at HF [40,41,42], MP2 [43,44,45,46,47], MP4 [48,49] and CCSD(T) [50,51] levels of theory and with basis sets of different sizes, thus the total energies are corrected to high-level theory and complete basis set [52,53]. Benchmark calculations for BDEs or enthalpies of formation were applied to different organic compounds [54,55,56]. The comparison shows that saving computer time dramatically decreases the accuracy of the composite method used. Benchmark studies predicted for the calculated BDEs the highest accuracy of the G4 method, with mean unsigned deviation or signed deviation being 2.44 kcal/mol and 0.36 kcal/mol, respectively [55]. It is therefore not surprising that this composite method also gives one of the best estimates of enthalpies of formation, although the computational costs of the G4 method are higher. Thus, for tens of $C_{x}$$H_{y}$$O_{z}$ molecules [54] the mean unsigned and signed deviations are 0.6 kcal/mol and −0.4 kcal/mol respectively. The benchmark studies performed with Xu et al. [55] are less accurate (although these were performed on a set of chlorinated/brominated compounds) for evaluation of enthalpies of formation, with mean unsigned and signed deviation of less than 2 kcal/mol and −2 kcal/mol, but the G4 method demonstrates the best performance overall.

### 4.4. GC-MS Measurement

The chemical composition of the gas inside the plasma reactor was analyzed using the GC-MS technique. The gas was sampled using the cold trap technique. The liquid nitrogen stainless steel trap (diameter of 15 mm, length of 165 mm, total volume of 116 ${\mathrm{cm}}^{3}$) was mounted at the reactor, as a side removable arm. The sampling time was 5 min and all gas products were subsequently analyzed by GC-MS. Gaseous samples in the liquid nitrogen trap were heated to the laboratory temperature. The reheating process may initiate thermal decomposition, oxidation, hydrolysis or other chemical reactions, especially in the presence of residual moisture or oxygen. It is assumed that due to the low temperatures at which the compounds are heated in the cold trap (i.e., room temperature in the cold trap and 100 °C at the inlet to the gas chromatograph), a change in molecular structure is unlikely. It has been found that overheating or uneven heating can lead to incomplete desorption or a change in the profiles of the compounds. This is due to the different boiling points of the compounds. It is assumed that due to the negative pressure inside the sampling device, the retention of volatile compounds in the cold trap is negligible in this case. However, these effects do not impact the identification of the compounds [57]. The resultant gas sample from the cold trap for GC-MS analysis was taken using a lock syringe just before being immediately analyzed. GC-MS analysis was carried out using an Agilent 7890B gas chromatograph (Agilent, Santa Clara, CA, USA) coupled to an Agilent 5977 quadrupole mass spectrometer (MS) (Agilent, Santa Clara, CA, USA). Separation was performed on a HP-5 ms column (30 m length, 0.25 mm internal diameter) using helium flow of 1.4 sccm as the carrier gas. Injection was at a 5:1 split and the injector temperature was 180 °C. The GC oven temperature was held for 4 min at 30 °C and then increased with a step of 15 °C $\min^{-1}$ to 120 °C. The MS was operated in an electron impact (70 eV) mode and scanned between 12–120 amu at approximately 11 scans per second.

### 4.5. OES Measurement

The spectra were measured using an Ocean Insights spectrometer, Flame T, FLMT10459 (Ocean Optics, Inc., EW Duiven, The Netherlands). The measurements were performed with an integration time of 1.5 s and 10 s for low signal observation. A 4 m long, 600 μm diameter quartz optical fiber was used for the measurements. The fiber was brought to the side of the gap between the discharge electrodes at a distance of 1 cm.

### 4.6. IMS-MS Measurement

The 2-methyl-2-oxazoline and 2-ethyl-2-oxazoline were studied using a home-made Ion Mobility Spectrometry (IMS) instrument well described in previous works [58,59,60]. The instrument was equipped with a point to plane corona discharge–atmospheric pressure chemical ionisation (CD-APCI) ion source, operated in the positive polarity and in reverse gas flow mode. The principle of IMS ion separation is based on the drift time of the ions in a homogeneous electric field along the drift tube filled with a drift gas. A voltage of 8 kV was applied to the whole drift tube of IMS to provide a drift field of 680 V ${\mathrm{cm}}^{-1}$. As a drift gas purified ambient air was used with a gas flow of 600 mL/min, while the sample flow was set to 50 mL/min. The micro-splitter valve (Supelco, Bratislava, Slovakia) controlled the sample flow rate was used and adjusted by a gas flow meter Platon NG (CT Platon, Saint-Etienne, France). The ion mobility scale has been calibrated using 2,6-di-tert-butylpyridine (Sigma-Aldrich, Bratislava, Slovakia) as a standard compound with the well-known reduced mobility of 1.41 ${\mathrm{cm}}^{2}V^{-1}s^{-1}$. The IMS was operated at a sub-atmospheric pressure of $6\times 10^{4}$ Pa and at 373 K. In the ionisation source, the reactant ions (RI) $H^{+}$·(H_2_O)_3,4_ were generated, which were used for the chemical ionisation of studied compounds. The IMS and MS spectra of the RI were measured on a standalone IMS and an orthogonal acceleration time-of-flight mass spectrometer (oa-TOF-MS).

### 4.7. FTIR Analysis of Film Extract

The FTIR spectra were measured using a spectrometer Bruker Vertex 80v (Bruker Optics GmbH & Co. KG, Ettlingen, Germany) with a 3 mm detector RT-DLaTGS and a KBr beamsplitter. The ATR diamond crystal was used for this measurement. A drop of solution was placed on the crystal and the spectra were measured. Also the FTIR spectra of pure water were measured.

### 4.8. Evaluation of Antibacterial Activity of Film Extract

The antibacterial activity of the extracts from polyoxazoline thin films deposited in previous studies [8,9] was tested using the EUCAST disk diffusion method (Kirby-Bauer method). The principle of the method consists of the diffusion of an antimicrobial substance into Mueller-Hinton agar with an inoculated microbial strain. If the growth of this strain is suppressed by an antimicrobial agent, a so-called inhibition zone will be created. The width of the inhibition zone serves as a measure of the antimicrobial effect of the tested solution. 100 μL sample was pipetted into an 8 mm diameter agar well, creating 2 sample wells on each dish. All samples were tested in triplicate. Then the dishes with all the samples were incubated at 35 °C. The bacterial inoculum concentration was $10^{8}$ CFU/mL (OD 0.5 Mac Farland).

## Figures and Tables

**Table 1:** Electron ionization of 2-methyl-2-oxazoline.

m (a.u.)	Cation	Neutral	AE G4 (eV)	AE Exp. (eV)
85	$C_{3}$$H_{4}$NO-C$H_{3}^{+}$	–	9.14	9.09
84	OC$H_{2}$CHNC-C$H_{3}^{+}$	H	10.44	9.90
56	C$H_{3}$-CH=N-C$H_{2}^{+}$	CH=O	10.19	10.11
55	C$H_{3}$-C=N-C$H_{2}^{+}$	C$H_{2}$=O	10.14	10.10
45	C$H_{3}$-CH-O$H^{+}$	C$H_{2}$-C≡N	10.18	10.59
44	C$H_{3}$-CH-$O^{+}$	${\mathrm{CH}}_{3}$-C≡N	10.57	10.50
43	C$H_{3}$-C=$O^{+}$	${\mathrm{CH}}_{2}$=C=N + $H_{2}$	10.81	10.40

**Table 2:** Electron ionization of 2-ethyl-2-oxazoline.

m (a.u.)	Cation	Neutral	AE G4 (eV)	AE Exp. (eV)
99	$C_{3}$ $H_{4}$ ${\mathrm{NO-C}}_{2}$ $H_{5}^{+}$	–	9.03	9.15
98	OC$H_{2}$CHNC-$C_{2}$$H_{5}^{+}$	H	10.37	10.40
70	C$H_{3}$-N=C-$C_{2}$$H_{5}^{+}$	CH=O	9.54	10.00
69	C$H_{2}$=N-C-$C_{2}$$H_{5}^{+}$	C$H_{2}$=O	10.04	10.40
54	${\mathrm{CH}}_{2}{\mathrm{=N-C=CH}}_{2}^{+}$	${\mathrm{CH}}_{2}$=O + ${\mathrm{CH}}_{3}$	12.46	12.23
45	${\mathrm{CH}}_{3}{\mathrm{-CH-OH}}^{+}$	${\mathrm{CH}}_{2}{\mathrm{-CH}}_{2}$-C≡N	10.46	10.60
44	${\mathrm{CH}}_{3}{\mathrm{-CH-O}}^{+}$	${\mathrm{CH}}_{3}{\mathrm{-CH}}_{2}$-C≡N	10.60	10.50
41	${\mathrm{CH}}_{3}{\mathrm{-C=N}}^{+}$	${\mathrm{CH}}_{3}{\mathrm{-CH}}_{2}$-CH=O	12.59	12.00

**Table 3:** Dissociation energies of 2-methyl-2-oxazoline.

Radicals	DE G4 (eV)
cyc(${\mathrm{OCH}}_{2}{\mathrm{CH}}_{2}$NC) + ${\mathrm{CH}}_{3}$	4.77
cyc(${\mathrm{OCH}}_{2}{\mathrm{CH}}_{2}$) + ${\mathrm{N-C-CH}}_{3}$	1.5
${\mathrm{OCH}}_{2}{\mathrm{CH}}_{2}$ + ${\mathrm{N-C-CH}}_{3}$	4.1

**Table 4:** Dissociation energies of 2-ethyl-2-oxazoline.

Radicals	DE G4 (eV)
cyc(${\mathrm{OCH}}_{2}{\mathrm{CH}}_{2}$NC)${\mathrm{-CH}}_{2}$ + ${\mathrm{CH}}_{3}$	3.61
cyc(${\mathrm{OCH}}_{2}{\mathrm{CH}}_{2}$NC) + $C_{2}{\mathrm{CH}}_{3}$	4.66
cyc(${\mathrm{OCH}}_{2}{\mathrm{CH}}_{2}$) + ${\mathrm{N-C-CH}}_{2}{\mathrm{-CH}}_{3}$	1.53
${\mathrm{OCH}}_{2}{\mathrm{CH}}_{2}$ + ${\mathrm{N-C-CH}}_{2}{\mathrm{-CH}}_{3}$	4.12

## Data Availability

The original contributions presented in this study are included in the article. Further inquiries can be directed to the corresponding author.
